# Gut microbiota differs between two cold-climate lizards distributed in thermally different regions

**DOI:** 10.1186/s12862-022-02077-8

**Published:** 2022-10-21

**Authors:** Jun-Qiong Chen, Lu-Wen Zhang, Ru-Meng Zhao, Hai-Xia Wu, Long-Hui Lin, Peng Li, Hong Li, Yan-Fu Qu, Xiang Ji

**Affiliations:** 1 Jiangsu Key Laboratory for Biodiversity and Biotechnology, College of Life Sciences, Nanjing Normal University, 210023 Nanjing, Jiangsu China; 2 Zhejiang Provincial Key Laboratory for Water Environment and Marine Biological Resources Protection, College of Life and Environmental Sciences, Wenzhou University, 325035 Wenzhou, Zhejiang China; 3 Hangzhou Key Laboratory for Ecosystem Protection and Restoration, College of Life and Environmental Sciences, Hangzhou Normal University, 311121 Hangzhou, Zhejiang China

**Keywords:** 16S rRNA gene sequencing, Cold-climate adaptation, Gut microbiota, *Phrynocephalus erythrurus*, *Phrynocephalus przewalskii*, Toad-headed lizard

## Abstract

**Background:**

The metabolic cold-climate adaption hypothesis predicts that animals from cold environments have relatively high metabolic rates compared with their warm-climate counterparts. However, studies testing this hypothesis are sparse. Here, we compared gut microbes between two cold-climate lizard species of the genus *Phrynocephalus* to see if gut microbiota could help lizards adapt to cold environments by promoting metabolism. We conducted a 2 species (*P. erythrurus* and *P. przewalskii*) × 2 temperatures (24 and 30 °C) factorial design experiment, whereby we kept lizards of two *Phrynocephalus* species at 24 and 30 °C for 25 d and then collected their fecal samples to analyze and compare the microbiota based on 16S rRNA gene sequencing technology.

**Results:**

The gut microbiota was mainly composed of bacteria of the phyla Proteobacteria, Firmicutes, Bacteroidetes, and Verrucomicrobia in both species (Proteobacteria > Firmicutes > Verrucomicrobiota in *P. erythrurus*, and Bacteroidetes > Proteobacteria > Firmicutes in *P. przewalskii*). Further analysis revealed that the gut microbiota promoted thermal adaptation in both lizard species, but with differences in the relative abundance of the contributory bacteria between the two species. An analysis based on the Kyoto Encyclopedia of Genes and Genomes revealed that the gut microbiota played important roles in metabolism, genetic information processing, cellular processes, and environmental information processing in both species. Furthermore, genes related to metabolism were more abundant in *P. erythrurus* at 24 °C than in other species ⋅ temperature combinations.

**Conclusion:**

Our study provides evidence that gut microbiota promotes thermal adaptation in both species but more evidently in *P. erythrurus* using colder habitats than *P. przewalskii* all year round, thus confirming the role of gut microbiota in cold-climate adaptation in lizards.

**Supplementary Information:**

The online version contains supplementary material available at 10.1186/s12862-022-02077-8.

## Background

Temperature is a major environmental factor that influences many biological aspects of organisms, including morphology [1], physiology [2], behavior [3], and distribution [4]. The metabolic cold adaption hypothesis or Krogh’s rule predicts that animals from cold environments have relatively high metabolic rates compared to those from warmer regions [5, 6]. High metabolic rates help animals balance the adverse effects of cold environments by accelerating their physiological processes in a relatively short time [5, 7]. Animals adapt to cold environments through physiological [5] and genomic adjustments [8]. For example, blackspotted topminnows (*Fundulus olivaceus*) with a northerly (colder) distribution have a higher metabolic rate than conspecifics with a southerly (warmer) distribution [5]. Another example is that adaptation to cold environments is mainly due to the physiological and biochemical capabilities in amphibians and reptiles that contribute to the development of cold tolerance [9]. However, the role of gut microbiota in cold adaptation is uncertain. Although several studies have demonstrated that temperature significantly influences the gut microbiota [10–12], studies on its role in the host’s cold adaptation are rare.

The mutualistic relationship between the host and its gut microbiota has been known for decades. Host animals provide suitable habitats and sufficient nutrients for the survival of gut microorganisms, which, in turn, contribute to nutrient absorption [13], intestinal homeostasis [14], and energy balance [15] of the host by encoding more genes than the host genome [16]. Thus, gut microorganisms play a crucial role in host survival [17] and adaptation [18, 19]. Temperature affects the composition and relative abundance of host gut microbiota [10–12]. Gut microbiota changes at low temperatures are more conducive to the survival and adaptation of host animals in cold climates [10, 14, 20]. In mice, the gut microbial changes in cold environments increase the gut absorptive surface area by lengthening the intestinal villi and microvilli, thus enhancing energy extraction [14]. In salamanders, cold temperature increases alpha diversity of gut microbial communities and results in improved digestive performance [21].

Reptiles are the first land vertebrates in the amniotic phylogeny and regulate their body temperate by selectively exploit external heat sources. Earlier studies suggest that gut microbiota promotes adaptation to various habitats in reptiles [22–26]. For example, it helps invasive red-eared slider turtles (*Trachemys scripta elegans*) colonize new habitats better than native Chinese three-keeled pond turtles (*Chinemys reevesii*) [25]. Another example is that the relative abundance of specific bacteria changes with altitude and is associated with adaptation to the Qinghai-Tibet Plateau in Qinghai toad-headed lizards (*Phrynocephalus vlangalii*) [26]. Therefore, understanding the coevolution of reptiles and their gut microbiota will reveal their complex environmental adaptation mechanism.

**Fig. 1:**
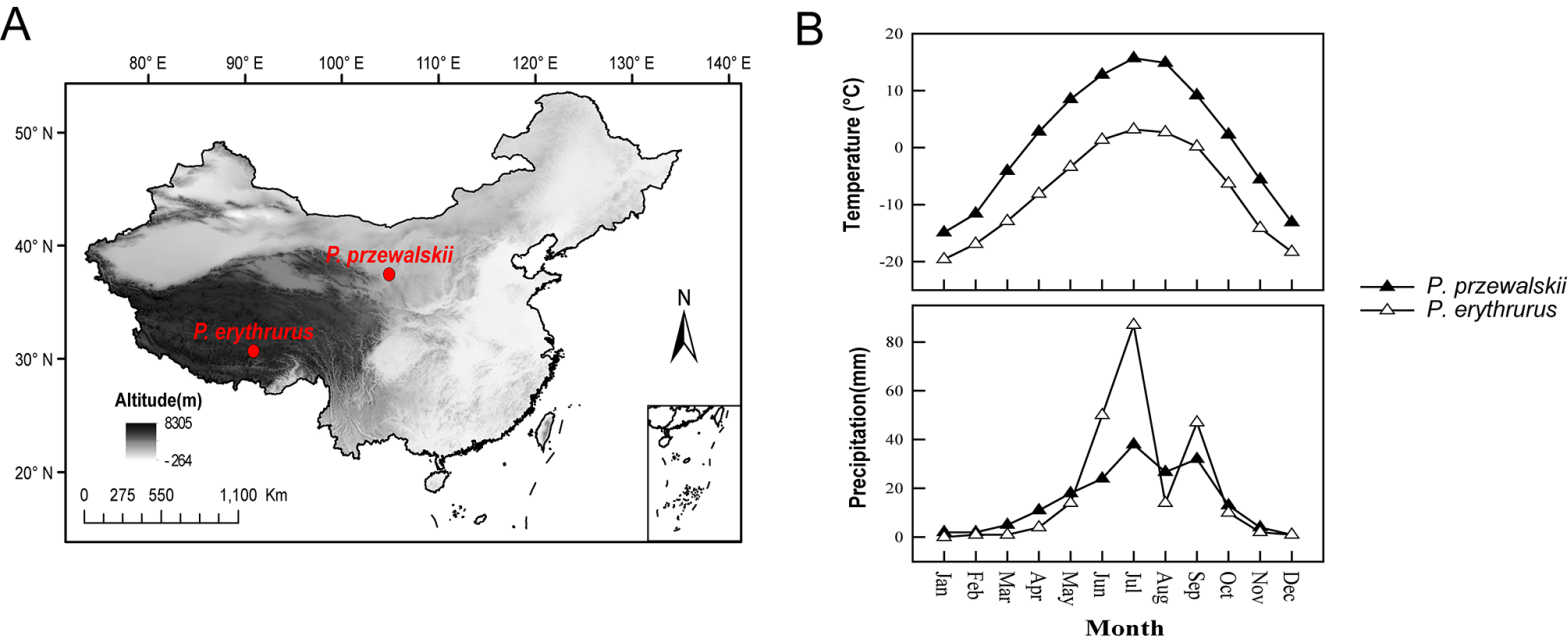
Map of China (**A**), showing geographical coordinates and monthly mean values for air temperature and rainfall (**B**) of the two localities, from which adult females of *P. erythrurus* (△, Namucuo, Tibet) and *P. przewalskii* (▲, Zhongwei, Ningxia) were collected

Toad-headed lizards of the reproductively bimodal genus *Phrynocephalus* (Agamidae) have a Palaearctic distribution and occur in desert, arid or semi-arid regions in Central and West Asia and North-Northwest China [27]. *Phrynocephalus erythrurus* (viviparous) and *P. przewalskii* (oviparous) studied herein have a strong research foundation in the fields of physiology [2], life history characteristics [28] and molecular biology [29]. Both species occur in cold-climate regions, with *P. erythrurus* using colder habitats than *P. przewalskii* all year round (Fig. 1). An earlier study on *P. przewalskii* reveals a higher digestive coefficient and assimilation efficiency at low temperature (25 °C) than at medium (33 °C) and high (39 °C) temperatures [2], indicating the potential role of gut microbiota in their cold-climate adaptation. However, no studies have elucidated the role of the gut microbiota in cold-climate adaptation in *Phrynocephalus* lizards.

Here, we studied the gut microbiota of *P. erythrurus* and *P. przewalskii* and their role in host adaptation to prolonged cold environments. We maintained lizards of these two *Phrynocephalus* species at two temperatures (24 and 30 °C) to see if there were taxonomic and functional differences in gut microbiota between the two lizards at different temperatures. We hypothesized that gut microbiota would help lizards adapt to cold environments and, specifically, there would be more gut microbial genes related to metabolic function in the species (*P. erythrurus*) using colder habitats than the other one (*P. przewalskii*) all year round.

## Methods

### Animal collection and maintenance

In late July 2020, we collected by hand 14 adult females of *P. erythrurus* [45−56 mm snout-vent length (SVL); 3.5−7.0 g body mass] from Namucuo (30°69’N, 90°87’E; ~4720 m elevation), Tibet, and 14 adult females of *P. przewalskii* (48−56 mm SVL; 4.6−6.7 g body mass) from Zhongwei (37°46’N, 104°87’E; ~1220 m elevation), Ningxia. Both places are dry and cold, with Namucuo being colder than Zhongwei all year round (Fig. 1) and unsuitable for the survival of oviparous *Phrynocephalus* lizards [30]. The 28 female lizards were brought by train to our laboratory at Nanjing Normal University, where 4−5 lizards of the same species were housed together in a plastic cage [500 × 400 × 360 mm (length × width × height)] with a sand substrate (~ 150 mm depth) and pieces of ceramic tiles for shade and shelter. These cages were placed in a room at temperatures varying from 24−30 °C for about five weeks before we started the experiment in September. During this time interval, thermoregulatory opportunities were provided during daytime (07:00−19:00 h) using a 60 W incandescent lamp suspend, and mealworms (larvae of *Tenebrio molito*r) and water enriched with vitamins and minerals were provided *ad libitum*.

### Experiment design and sample collection

We conducted a two temperatures (24 and 30 °C) ⋅ two species (*P. erythrurus* and *P. przewalskii*) factorial design experiment in late August 2020. We chose 24 °C because, although cold-climate *Phrynocephalus* lizards never experience ambient temperatures with a daily mean higher than 24 °C, the temperature is too low for them to display normal physiological and behavioural activities. For example, *Phrynocephalus* lizards do not eat at temperatures lower than 24 °C [2, 30], and their embryos cannot complete development at temperatures lower than 24 °C [1, 31, 32]. We chose 30 °C because it is about 1 °C above the lower limit of body temperatures selected by *Phrynocephalus* lizards in laboratory thermal gradients [2, 33, 34].

Prior to the experiment, two AAPS (artificial atmospheric phenomena simulator) rooms and all experimental items were disinfected with alcohol wipes and an ultraviolet lamp. We individually weighed (to the nearest 0.01 on a Mettler balance) and measured (for SVL to the nearest 0.1 mm using Mitutoyo digital calipers) lizards, and then housed each lizard in a 350 × 240 × 190 mm plastic cage. We placed 12 lizards (six of each species) in an AAPS room at 24 ± 1 °C and 16 (eight of each species) in an AAPS room at 30 ± 1 °C. Fluorescent lights in both rooms were switched on at 07:00 h and off at 19:00 h throughout the experiment. Mean values for body mass and SVL did not differ between the two temperature treatments in both species (*F*_1, 12_ < 0.71 and *P* > 0.41 in all cases). Mealworms sterilized with an ultraviolet lamp 1 h before feeding and distilled water were provided *ad libitum* for 25 d. Feces of each lizard were collected in sterile centrifuge tubes under sterile conditions at two intervals, one between day 9 and day 12 (0.05 ± 0.01 g for *P. przewalskii* and 0.06 ± 0.02 g for *P. erythrurus* at 24 °C, 0.20 ± 0.04 g for *P. przewalskii* and 0.13 ± 0.03 g for *P. erythrurus* at 30 °C), and another between day 21 and day 25 (0.08 ± 0.02 g for *P. przewalskii* and 0.06 ± 0.03 g for *P. erythrurus* at 24 °C, 0.18 ± 0.05 g for *P. przewalskii* and 0.18 ± 0.04 g for *P. erythrurus* at 30 °C). All 56 fecal samples were individually labeled and stored at −80 °C for DNA extraction. All lizards were released to the sites where they were collected in late September 2020.

### DNA extraction, PCR amplification and sequencing of fecal samples

The total DNA was extracted from the fecal samples using the E.Z.N.ATM Mag-Bind Soil DNA Kit (OMEGA Bio-Tek, Norcross, GA, USA) according to the manufacturer’s instructions. All samples were used in subsequent PCR amplification, and no negative controls were used to detect the bacterial DNA contamination. The DNA quality was assessed on an agarose gel (1.0%), and the concentration was detected using a Qubit3.0 DNA detection kit (Thermo Fisher Scientific, Shanghai, China). The concentration of DNA extracted from fecal samples was 1.73 ± 0.65 (0.11−13.80) ng/µL in *P. erythrurus*, and 1.38 ± 0.51 (0.10−11.90) ng/µL in *P. przewalskii*. The V3-V4 region of the bacterial 16S rRNA gene was amplified by polymerase chain reaction (PCR) using the gene-specific primers 341F (5’-CCTACGGGNGGCWGCAG-3’) and 805R (5’-GACTACHVGGGTATCTAATCC-3’). The first round of PCR was conducted in a 30 µL reaction mixture consisting of 15 µL of Hieff Robust PCR Master Mix (2×), 1 µL of each primer (10 µM), 10−20 ng of genomic DNA, and 9−12 µL of ddH_2_O. The thermal cycling conditions were set as follows: initial denaturation at 94 °C for 3 min, followed by 5 cycles of denaturation at 94 °C for 30 s, annealing at 45 °C for 20 s and extension at 65 °C for 30 s, 20 cycles of denaturation at 94 °C for 20 s, annealing at 55 °C for 20 s, and extension at 72 °C for 30 s, and 5 min at 72 °C. The second round of PCR was conducted using the first-round PCR amplicon as the template. The six-base recognition sequence (barcodes) and Illumina bridge PCR compatible primers were introduced in the second PCR amplification after the first. The PCR mixture used was the same as the first round. The thermal cycling conditions were set as follows: denaturation at 95 °C for 3 min, followed by 5 cycles of denaturation at 94 °C for 20 s, annealing at 55 °C for 20 s and an extension at 72 °C for 30 s, and a final extension at 72 °C for 5 min. We used negative and positive controls in each batch of samples to check DNA contamination and integrity. The positive control was composed of a standard PCR reaction mixture containing 10 ng of *Escherichia coli* genomic DNA instead of sample, and the negative control contained sterile water instead of sample. The PCR products electrophoresed on a 2.0% agarose gel were purified, and the concentration was detected using a Qubit3.0 DNA detection kit (Thermo Fisher Scientific). Subsequently, 10 ng of the PCR product (600 pmol) was used from each sample for pyrosequencing on an Illumina NovaSeq 6000 system at Shanghai Sangon Biotech., China.

### Data process and standardization

We used the Quantitative Insights Into Microbial Ecology 2 (QIIME2) platform [35] to process the raw sequencing reads. Subsequently, we used the DADA2 package [36] to filter and trim the low-quality reads to produce paired-end reads, and merged these paired-end reads to obtain the amplicon sequence variants (ASVs). The taxonomy was assigned to the ASVs using the pre-formatted SILVA 138 SUURef NR99 ASVs full-length reference sequences following the q2-fragment-classifier method in QIIME2. These sequences were submitted to the National Genomics Data Center (NGDC) GSA database (accession number CRA004548). ASVs with the number of ASVs greater than 10 in at least two samples were retained for further analysis to avoid the effect of low read numbers on the results using QIIME2. Finally, the ASV abundance was standardized based on the sample with the least ASVs for alpha and beta diversity analysis.

### Comparison and diversity of gut microbiota

The alpha diversity matrices, including community richness (Chao1 index), community diversity (Shannon diversity index), and community evenness (Simpson’s evenness index), were calculated in QIIME2. We used the R software to visualize these indices. Further, we performed principal coordinates analysis (PCoA) and analysis of similarity (ANOSIM) to determine the differences in the structure (beta diversity) and communities of the gut microbiota among the different species ⋅ temperature combinations. The cluster analysis was performed using Bray-Curtis similarity to explore the similarities in the gut community composition between the samples. ANOSIM based on the Bray-Curtis distance metrics with 999 permutations was further conducted to analyze the differences across the four groups (2 species × 2 temperatures) [37]. We performed a betadisper analysis using package vegan in R to test sample heterogeneity before performing ANOSIM.

Subsequently, we used linear discriminant analysis effect size (LEfSe) [38] to compare the microbial abundances, from the phylum to family levels, among the four groups. Then, we used linear discriminatory analysis (LDA) to evaluate the effect size for each selected classification. Here, we only used the dominant bacterial taxa with a log LDA score > 3.5 (over 3.5 orders of magnitude). We used Kruskal-Wallis test to determine the bacteria detected by LDA that had a significantly higher relative abundance in the four groups.

### Gene function prediction

Further, the gene functions based on 16S rRNA sequences were predicted using PICRUSt2 based on the Kyoto Encyclopedia of Genes and Genomes (KEGG) database [39], and the genes were classified and allocated to the corresponding KEGG pathways [40]. Gene functions are stored in the KEGG Orthology (KO) database, where each KO is defined as a functional homology of genes and proteins. KOs in each pathway were counted to assess their relative abundance in each species ⋅ temperature combination. The first three KO levels are corresponded to the KEGG functional categories and the fourth level corresponds to the KO ID. We performed PCoA and ANOSIM to determine the differences in the gene functions (KOs) of the gut microbiota using the method described above. Further, we used LEfSe to compare the differential relative abundance of the KEGG gene functions from level 1 to level 3 among the different species ⋅ temperature combinations. We performed LDA analysis to assess the effect size for each selected level. Here, we only used the functional category with a log LDA score > 2.5. Again, we used Kruskal-Wallis test to determine the bacterial functions detected by LDA that had a significantly higher relative abundance in the four groups.

### Statistical analysis

A series of preliminary analyses showed that neither alpha [one-way analysis of variance (ANOVA): *F*_1,10_ < 4.59, *P* > 0.05] nor beta [analysis of similarities (ANOSIM): *R* < 0.14, *P* > 0.14) diversity differed between samples collected at the two time intervals (Day 9−12 and Day 21−24) within each species ⋅ temperature combination. Therefore, we pooled the data collected at the two time intervals. We used two-way analysis of variance (ANOVA) to test the effects of species, temperature, and their interaction on alpha diversity and the relative abundance of the gene functions. All values were presented as mean ± standard error (SE), and the differences were considered statistically significant at α < 0.05.

## Results

### Fecal bacterial sequencing and AVSs

A total of 1,999,484 and 2,072,977 high-quality reads were obtained from *P. erythrurus* and *P. przewalskii*, respectively, after quality control. SRA accession number and group information are given in Table S1. The rarefaction curves (Fig. S1) and species accumulation curves (Fig. S2) increased with sample size and attained a precise saturation level, indicating adequate sampling and sufficient sequencing depth in the experiment. Further, based on 99% ASVs full-length sequence reference database, we identified 992 ASVs from the 14 *P. erythrurus* samples (44–265 ASVs per sample) and 1518 ASVs from the 14 *P. przewalskii* samples (147–371 ASVs per sample) (Table S2). Among these ASVs, 1778 from the 56 fecal samples of the two species were taxonomically assigned to 15 phyla, 22 classes, 63 orders, 102 families, and 191 genera.

### Composition and abundance of gut microbiota

**Fig. 2:**
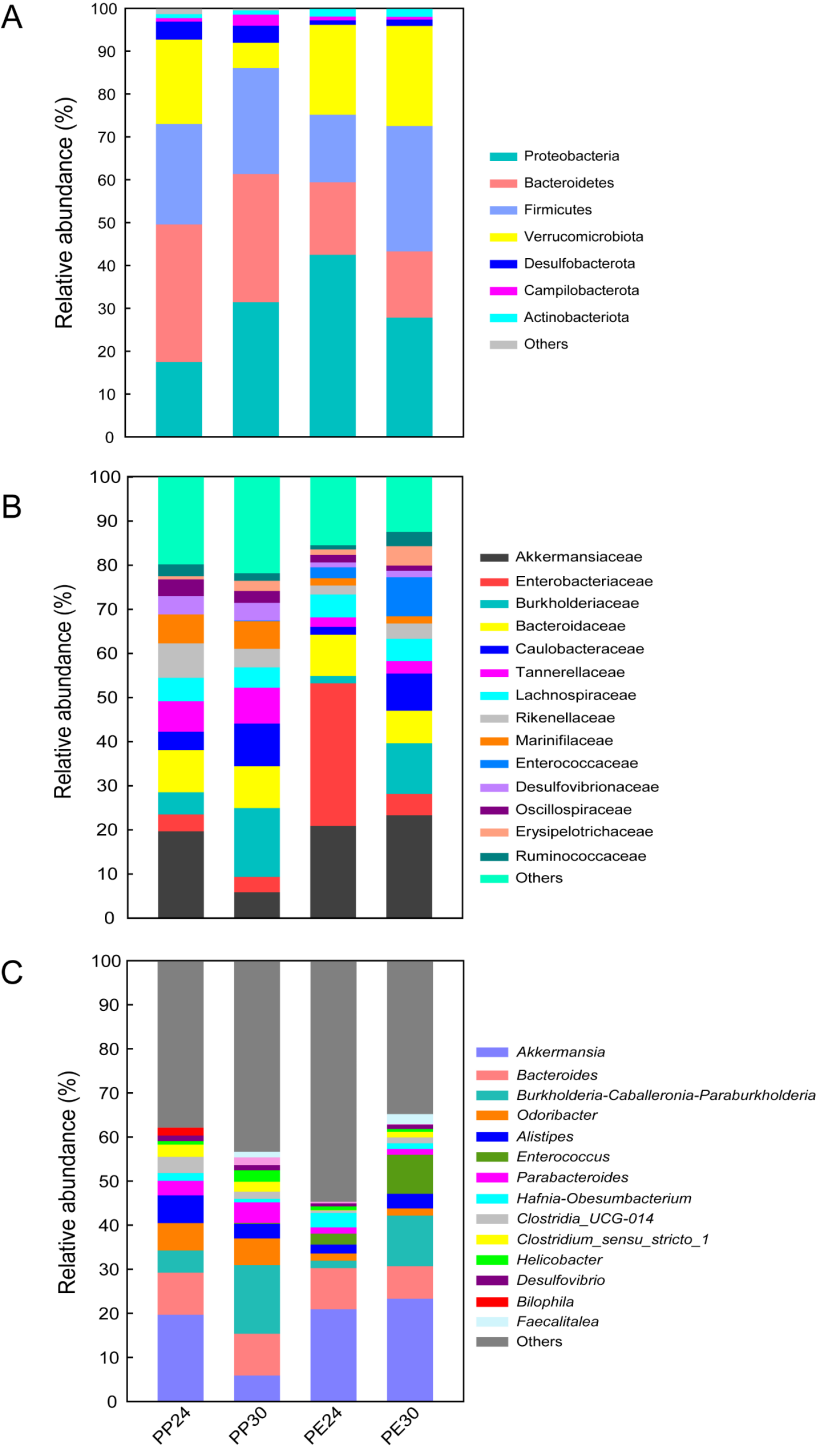
The relative abundance of the gut microbiota at the phylum (**A**), family (**B**), and genus (**C**) levels in each of four groups ( 2 species × 2 temperatures). One color indicates one taxon in each plot, and the color for “Others” indicates all other taxa not listed in each plot. PP24: *P. przewalskii* at 24 °C; PP30: *P. przewalskii* at 30 °C; PE24: *P. erythrurus* at 24 °C; PE30: *P. erythrurus* at 30 °C

We first analyzed individual bacterial taxa with a relative abundance higher than 3%. In the two species as a whole, Proteobacteria (29.8 ± 3.6%), Firmicutes (23.8 ± 1.5%), Bacteroidetes (23.5 ± 1.8%), and Verrucomicrobia (17.1 ± 2.1%) were the top four dominant phyla (Fig. 2 A), Akkermansiaceae (17.1 ± 2.1%), Enterobacteriaceae (10.1 ± 2.5%), Burkholderiaceae (9.2 ± 1.8%), Bacteroidaceae (8.9 ± 0.8%), Caulobacteraceae (6.5 ± 1.3%), Tannerellaceae (5.1 ± 0.6%), and Lachnospiraceae (5.0 ± 0.5%) were the top seven dominant families (Fig. 2B), and *Akkermansia* (17.1 ± 2.1%), *Burkholderia-Caballeronia-Paraburkholderia* (9.2 ± 1.8%), and *Bacteroides* (8.9 ± 0.8%) were the top three dominant genera (Fig. 2 C). However, the abundance order of the dominant gut microbial taxa differed between the two species. The top two dominant bacterial phyla in *P. przewalskii* were Bacteroidetes (30.8 ± 0.1%) and Proteobacteria (25.5 ± 4.4%), followed by Firmicutes (24.2 ± 1.9%), Verrucomicrobiota (11.8 ± 2.0%), and Desulfobacterota (4.1 ± 0.5%), while those in *P. erythrurus* were Proteobacteria (34.1 ± 5.5%) and Firmicutes (23.5 ± 2.2%), followed by Verrucomicrobiota (22.3 ± 3.4%) and Bacteroidetes (16.1 ± 1.9%) (Fig. 2 A). Akkermansiaceae (17.1 ± 0.02%) was the most abundant bacterial family in both species, followed by Burkholderiaceae (11.1 ± 2.5%), Bacteroidaceae (9.5 ± 1.0%), Tannerellaceae (7.6 ± 0.9%), and Caulobacteraceae (7.3 ± 2.0%) in *P. przewalskii* and Enterobacteriaceae (16.6 ± 4.4%), Bacteroidaceae (8.2 ± 1.2%), Burkholderiaceae (7.3 ± 2.4%), and Enterococcaceae (6.1 ± 1.9%) in *P. erythrurus* (Fig. 2B). Following the top dominant genera (*Akkermansia*, *Burkholderia-Caballeronia-Paraburkholderia*, and *Bacteroides*), the abundant genera were *Odoribacter* (4.6 ± 0.8%), *Alistipes* (4.1 ± 0.6%), and *Parabacteroides* (2.7 ± 0.5%) were abundant in *P. przewalskii*, and *Enterococcus* (3.4 ± 1.7%) in *P. erythrurus* (Fig. 2 C).

### Alpha and beta diversity of gut microbiota

**Fig. 3:**
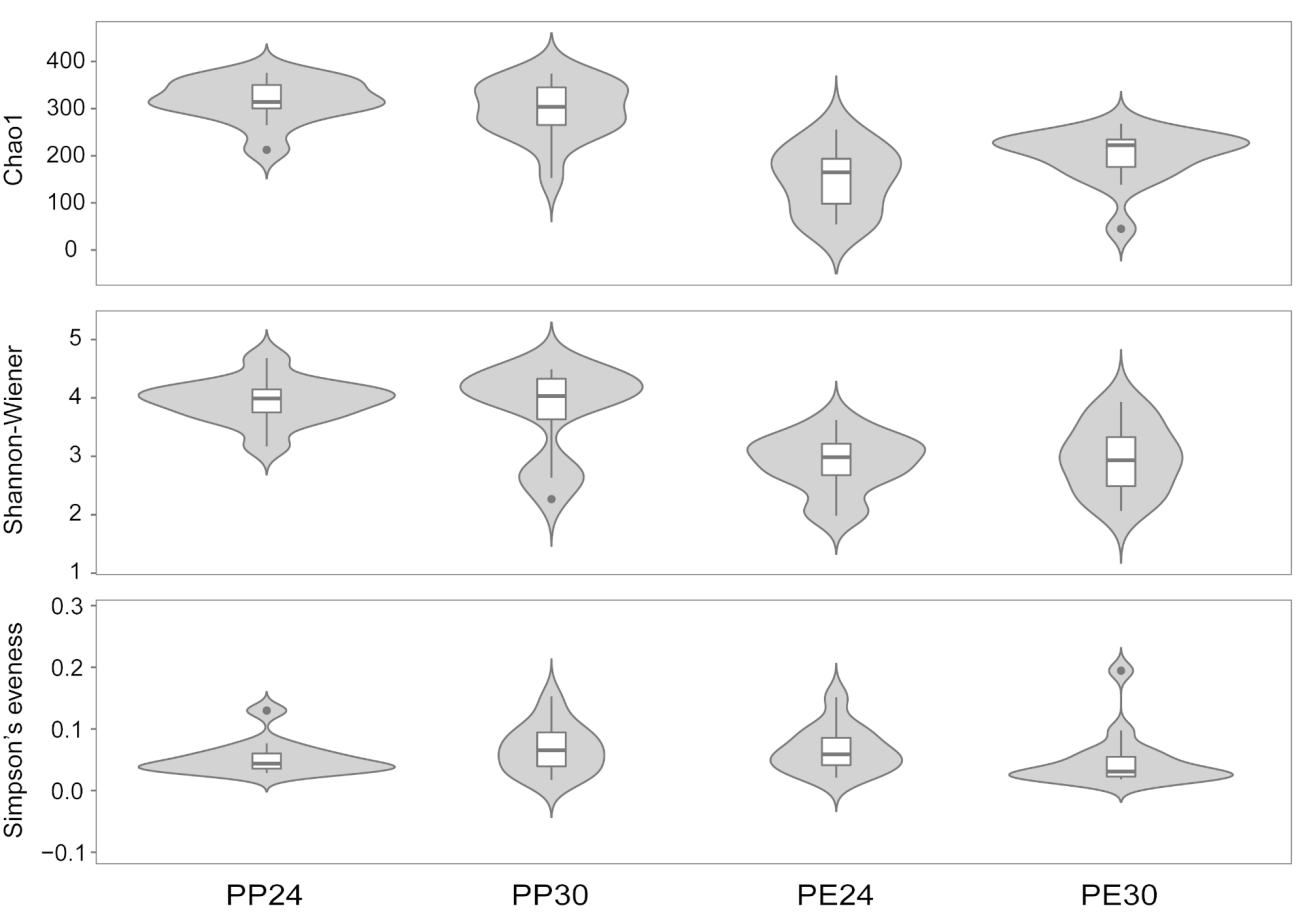
Chao1 index, Shannon-Weiner index, and Simpson’s eveness index of gut microbiota in each of four groups (2 species × 2 temperatures). See Fig. 2 for the definition of each combination

**Fig. 4:**
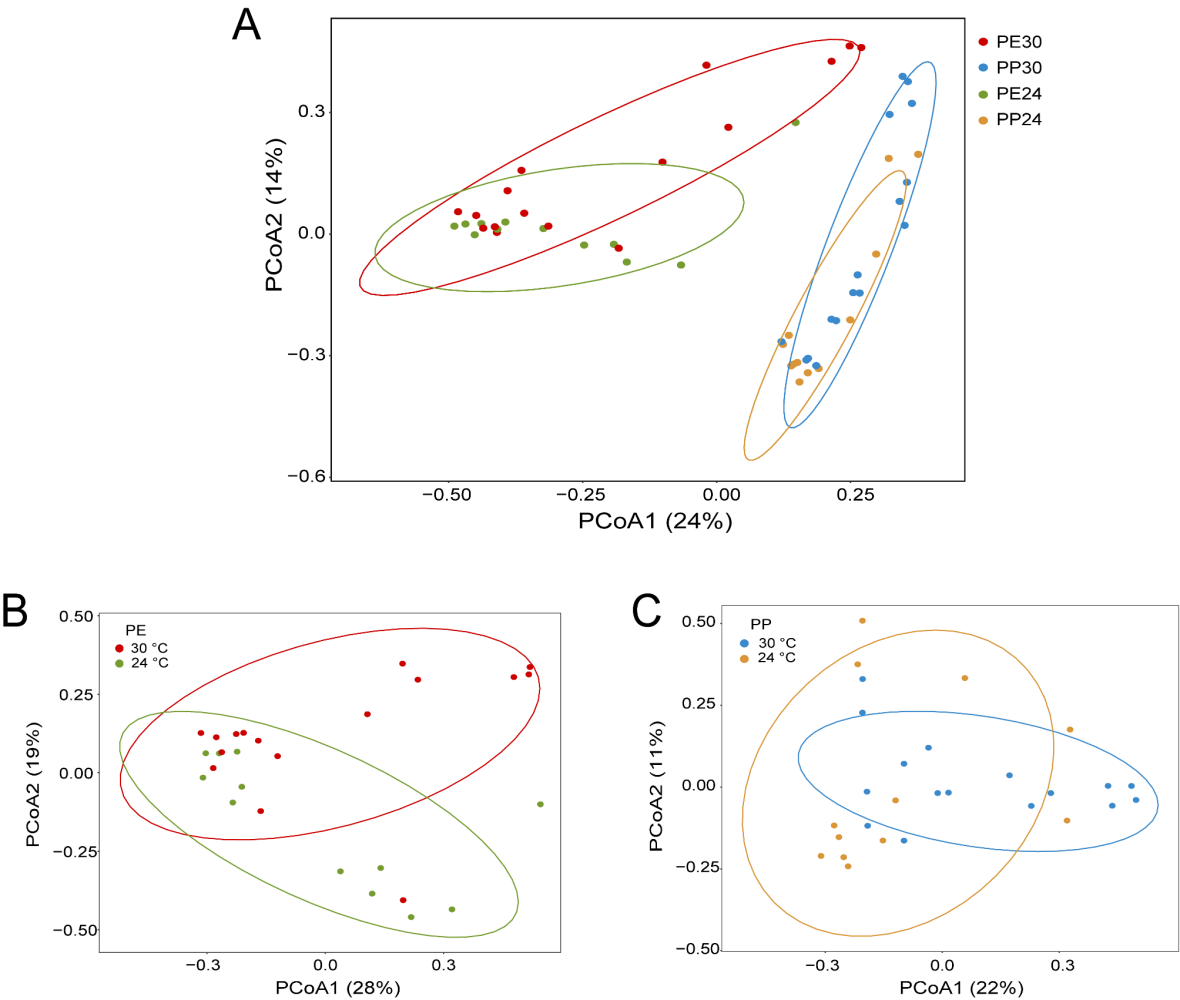
Results of principal coordinates analysis of Bray-Curtis distance matrix for gut microbial diversity in each of four groups (2 species × 2 temperatures) (**A**), *P. erythrurus* at 24 and 30 °C (**B**), and *P. przewalskii* at 24 and 30 °C (**C**). One color indicates one combination. See Fig. 2 for the definition of each combination

From results of two-way ANOVA we knew the following. First, Chao1 (*F*_1,52_ = 54.45, *P* < 0.0001) and Shannon (*F*_1,52_ = 36.44, *P* < 0.0001) indexes differed significantly between *P. erythrurus* and *P. przewalskii*; however, Simpson’s eveness index showed no difference (*F*_1,52_ = 0.09 *P* = 0.76). Second, Chao1 and Shannon diversity indexes were higher in *P. przewalskii* than in *P. erythrurus* (Fig. 3). Third, temperature treatment (*F*_1,52_ < 0.92 and *P* > 0.34) and the interaction between species and temperature (*F*_1,52_ < 2.52 and *P* = 0.11) showed no significant effect on these three diversity indexes. Subsequent betadisper analysis showed the differences in variability of bacterial community among the four species ⋅ temperature combinations were homogenous (*F*_3,52_ = 0.86 *P* = 0.47). PCoA showed a significant separation in gut microbial beta diversity among the four species ⋅ temperature combinations (Table 1; Fig. 4 A), with PCo1 and PCo2 respectively accounting for 24% and 14% of the total variance (Fig. 4 A). Given that there was a clear microbiota separation between the two species, we performed another PCoA for each species. We found a significant separation in bacterial composition between the two temperature treatments in each species, and that PCo1 and PCo2 together explained 47% of the total variance in *P. erythrurus* (Table 1; Fig. 4B) and 33% of the total variance in *P. przewalskii* (Table 1; Fig. 4 C).

**Table 1:** The ANOSIM results used to evaluate the effect of the defined categories based on Bray-Curtis distances

Categories	*df*	*F*	*R*	*P*
All groups	3	6.214	0.581	0.001
*P. erythrurus*	1	2.884	0.194	0.010
*P. przewalskii*	1	2.174	0.176	0.009

The categories included all groups (= four species ⋅ temperature combinations), *P. erythrurus*, and *P. przewalskii*

**Fig. 5:**
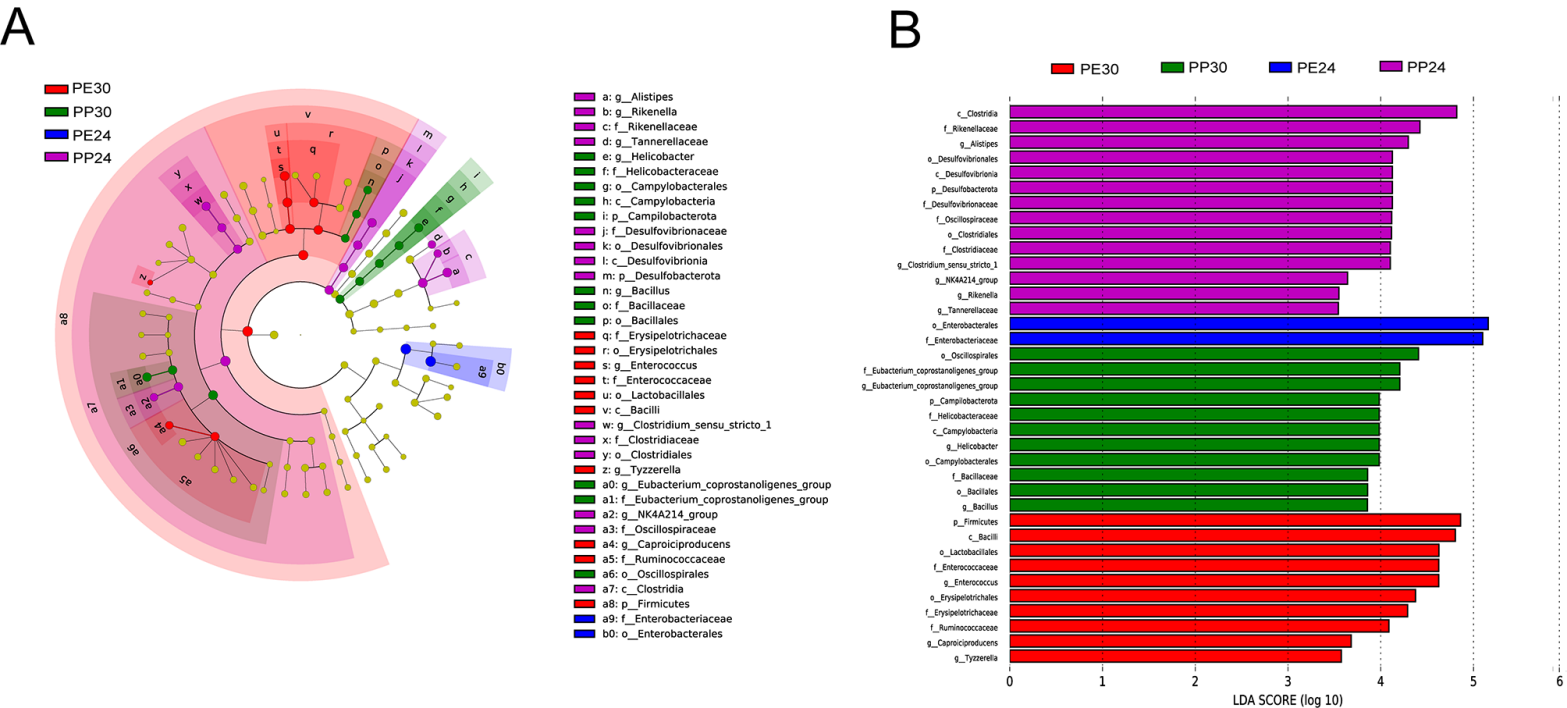
Differences in bacterial taxa among the four groups (2 species × 2 temperatures) determined by LEfSe (**A**). LDA scores reflect the differences in relative abundance among four combinations (**B**). Letters p, c, o, f, and g indicate phylum, class, order, family, and genus, respectively. See Fig. 2 for the definition of each combination

Furthermore, the LEfSe and LDA analyses revealed that Enterobacteriaceae (*LDA* = 5.10, *P* = 0.001) was the unique bacterial family in *P. erythrurus* at 24 °C (Fig. 5), while the family Erysipelotrichaceae (*LDA* = 4.29, *P* = 0.002) and the genera *Caproiciproducens* (*LDA* = 3.68, *P* < 0.0001), *Tyzzerella* (*LDA* = 3.58, *P* = 0.04) and *Enterococcus* (*LDA* = 4.62, *P* = 0.0002) were unique at 30 °C (Fig. 5). In *P. przewalskii*, the family Desulfovibrionaceae (*LDA* = 4.13, *P* = 0.0003) and the genera *Alistipes* (*LDA* = 4.30, *P* = 0.03), *Tannerellaceae* (*LDA* = 3.54, *P* = 0.0003), *Rikenella* (*LDA* = 3.55, *P* < 0.0001), *NK4A214 group* (*LDA* = 3.64, *P* = 0.0001), and *Clostridium sensu stricto 1* (*LDA* = 4.10, *P* = 0.0007) were unique at 24 °C (Fig. 5), while the genera *Eubacterium coprostanoligenes group* (*LDA* = 4.21, *P* < 0.0001), *Bacillus* (*LDA* = 3.86, *P* = 0.0003), and *Helicobacter* (*LDA* = 3.98, *P* = 0.004) were unique at 30 °C (Fig. 5). Results of Kruskal-Wallis test showed that the relative abundance of most bacterial taxa mentioned above differed significantly among different groups, with exceptions including bacteria of the classes Campylobacteria and Desulfovibrionia, the order Campylobactera, and the genera *Alistipes*, *Tannerellaceae*, *Enterococcus*, *NK4A214_group*, *Rikenella*, *Caproiciproducens*, *Tyzzerella* and *Bacillus* (Table S3).

### Gene function predication

**Fig. 6:**
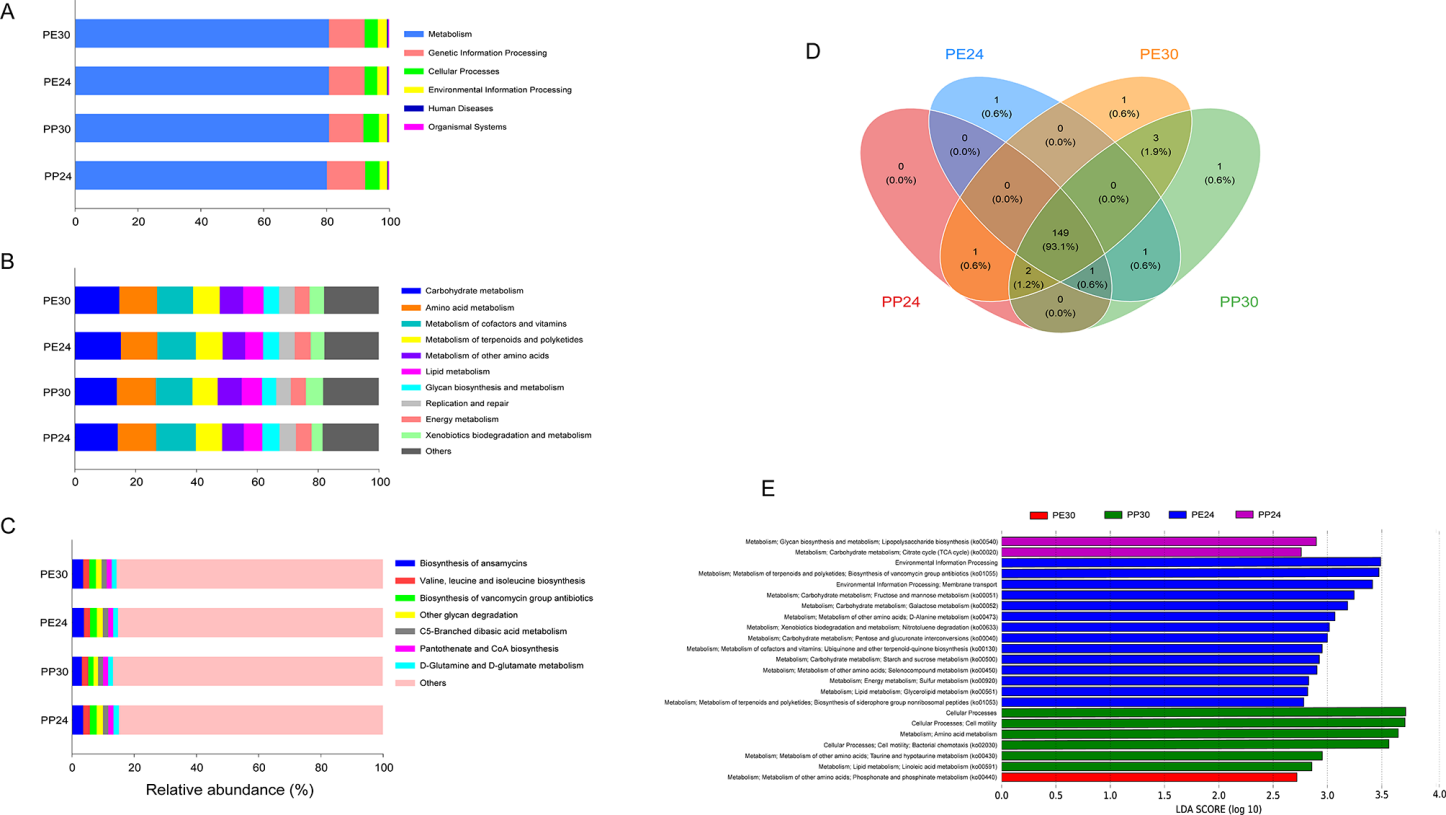
Relative abundance of gene functions at the first (**A**), second (**B**), and third (**C**) levels in the gut microbiota of two lizard species, and the Venn diagram showing the functional genes among fecal samples from four species × temperature combinations (**D**). LDA scores reflect the differences in relative abundance among the four groups (2 species × 2 temperatures) (**E**). One color indicates one gene function in each plot, with detailed descriptions shown on the right side of each plot. The colors for “Others” in plots B and C indicate all other gene functions not listed in these two plots. See Fig. 2 for the definition of each combination

Finally, the gene functions were predicted based on 16S RNA data of the 56 fecal samples. The analysis indicated that genes related to metabolism (80.6 ± 0.1%) were the most predominant at the top level in both species (Fig. 6 A). The other three major functional categories at the top level were genetic information processing (11.4 ± 0.2%), cellular processes (4.5 ± 0.1%), and environmental information processing (2.8 ± 0.08%) (Fig. 6 A). The metabolism-related functional category included carbohydrate metabolism (14.4 ± 0.1%), amino acid metabolism (12.5 ± 0.08%), metabolism of cofactors and vitamins (12.3 ± 0.2%), metabolism of terpenoids and polyketides (8.6 ± 0.1%), metabolism of other amino acids (7.6 ± 0.1%), lipid metabolism (6.4 ± 0.1%), glycan biosynthesis and metabolism (5.2 ± 0.2%), energy metabolism (5.0 ± 0.04%), and xenobiotics biodegradation and metabolism (4.7 ± 0.3%) as the predominant ones at the second level in both species (Fig. 6B). Other functional categories with a relative abundance greater than 3% included replication and repair (5.1 ± 0.1%) at the second level and genetic information processing at the first level in both species (Fig. 6B). At the third level, the most abundant gene functions in the four groups or 2 species ⋅ 2 temperature combinations were biosynthesis of ansamycins (3.5 ± 0.1%), valine, leucine, and isoleucine biosynthesis (2.0 ± 0.02%), and biosynthesis of vancomycin group antibiotics (2.0 ± 0.07%) (Fig. 6 C).

Furthermore, 160 known KEGG genes were identified based on 16S rRNA sequences in the two species. Venn diagram showed that most of the KOs were common in the different species × temperature combinations (Fig. 6D); 154 KOs were shared between the two species, and 149 between the 24 and 30 °C treatments in *P. erythrurus*, and 152 between the two temperature treatments in *P. przewalskii*. Although there were no significant differences in gene function variability among the four groups (*F*_3,52_ = 0.82 *P* = 0.49), PCoA could not separate the different groups based on the KOs (*r* = 0.07, *F*_3,52_ = 2.35 *P* = 0.05; Fig. S3).

The symbolic gene functions at 24 °C in *P. erythrurus* were related to metabolism, including phosphonate and phosphinate metabolism (ko00440; *LDA* = 3.00, *P* = 0.0009), fructose and mannose metabolism (ko00051; *LDA* = 3.25, *P* = 0.001), galactose metabolism (ko00052; *LDA* = 3.18, *P* = 0.02), starch and sucrose metabolism (ko00500; *LDA* = 2.92, *P* = 0.03), sulfur metabolism (ko00920; *LDA* = 2.83, *P* = 0.0006), glycerolipid metabolism (ko00561; *LDA* = 2.82, *P* = 0.002), ubiquinone and other terpenoid-quinone biosynthesis (ko00130; *LDA* = 2.95, *P* = 0.0009), selenocompound metabolism (ko00450; *LDA* = 2.90, *P* = 0.0006), D-alanine metabolism (ko00473; *LDA* = 3.07, *P* = 0.001), biosynthesis of siderophore group nonribosomal peptides (ko01053; *LDA* = 2.78, *P* = 0.0001), biosynthesis of vancomycin group antibiotics (ko01055; *LDA* = 3.47, *P* = 0.02), and nitrotoluene degradation (ko00633; *LDA* = 3.02, *P* = 0.001), and environmental information processing, including membrane transport (*LDA* = 3.41, *P* = 0.009) (Fig. 6E). However, only ko00440 related to phosphonate and phosphinate metabolism (*LDA* = 2.72, *P* = 0.02) showed a unique function at 30 °C in *P. erythrurus* (Fig. 6E). Gene function related to metabolism, including TCA cycle (ko00020; *LDA* = 2.76, *P* = 0.01) and lipopolysaccharide biosynthesis (ko00540; *LDA* = 2.90, *P* = 0.007), were unique at 24 °C in *P. przewalskii* (Fig. 6E). Meanwhile, *P. przewalskii* at 30 °C had unique KOs related to cellular processes, including bacterial chemotaxis (ko02030; *LDA* = 3.57, *P* = 0.006), and metabolism-related functions, including amino acid metabolism (*LDA* = 3.65, *P* = 0.0003), linoleic acid metabolism (ko00591; *LDA* = 2.86, *P* = 0.003), and taurine and hypotaurine metabolism (ko00430; *LDA* = 2.95, *P* = 0.002) (Fig. 6E). Results of Kruskal-Wallis test showed that the relative abundance of all aforementioned functions except ko00440 differed significantly among the four groups (Table S4). In addition, *P. erythrurus* had higher abundance of gene functions related to metabolism at 24 °C than *P. przewalskii*.

## Discussion

Investigating taxonomic and functional differences in gut microbiota associated with cold-climate and/or high-altitude adaptation can contribute to uncovering the mechanisms of cold adaptation in animals. In this study, the alpha diversity of gut bacterial communities differed between the two species but not between the two temperature treatments (Fig. 3). Similarly, temperature does not significantly affect the gut bacterial alpha-diversity in cows [41] or tadpoles [42]. However, most studies spanning a wide range of animal taxa, including invertebrates and vertebrates [12, 14, 21−22, 43], have shown that the gut bacterial alpha-diversity overall increases at high temperatures; the opposite has been observed in fish [10]. Past studies generally conclude that the gut microbial diversity in animals is related to their genotype or habitat environment [23, 26].

Both species studied herein live in cold environments with great and unpredictable thermal fluctuations. Therefore, the gut microbiota might have adapted to drastic temperature fluctuations during evolution [21]. In the present study, the alpha diversity was significantly higher in *P. przewalskii* than in *P. erythrurus* (Fig. 3). This observation is consistent with the study on the Qinghai toad-headed lizard *P. vlangalii*, where the lowest gut microbial diversity is detected in the highest altitude (coldest) population [26]. However, these results are different from those reported for mammals. High-altitude mammals (e.g., yaks, sheep, and pika) have higher bacterial diversity than their low-altitude relatives (cattle, sheep, and pika) due to their higher energy demands in cold and hypoxic environments [44, 45]; however, the cause for these differences between reptiles and mammals remains unknown. In addition, many studies have shown that the host genotype causes differences in the alpha-diversity in different host taxa [13, 45, 46]. For example, the alpha diversity of gut microbiota differs significantly among four species of fish larvae reared under the same conditions, indicating the influence of the genetic background of a species on the alpha diversity [47]. Therefore, the differences in gut microbial diversity between the two *Phrynocephalus* lizards observed in this study suggest an association with their genetic background.

The gut microbiota plays a vital role in animal adaptation and evolution [23, 26]. Past studies have reported significant differences in the composition of gut microbes at different taxon levels in reptiles due to various factors, including host genotype [47], dietary type [48], habitat use [26], sex [49], and ontogenetic stage [42]. The present study also found significant differences in the relative abundance of gut bacteria at different taxon levels among the different species and temperature combinations (Figs. 2 and 5). The top two dominant phyla of gut microbiota in *P. erythrurus* were Proteobacteria and Firmicutes (Fig. 2), consistent with *P. vlangalii* [26] and the marine iguana *Amblyrhynchus cristatus* [23]. Proteobacteria degrades and ferments complex sugars and synthesizes vitamins to supply nutrients for their hosts [50]. Meanwhile, the Firmicutes phylum is associated with enzymes involved in fermentation and vitamin B biosynthesis [51]. Therefore, the higher relative abundance of Proteobacteria and Firmicutes in *P. erythrurus* presumably suggests their roles in improving the host energy absorption efficiency and utilization rate. Similar to the crocodile lizard *Shinisaurus crocodilurus* [52], the dominant gut bacterial phyla were Bacteroidetes and Proteobacteria in *P. przewalskii* (Fig. 2). Bacteria of the phylum Bacteroidetes might be responsible for the breakdown of the complex macromolecules [50]. The ratio of Firmicutes to Bacteroidetes is negatively correlated with mass gain [53]. The lower Firmicutes/Bacteroidetes ratio suggests that the gut microbiota could rapidly increase the host mass at low temperatures, which is conducive to the host’s adaptation to the long-term cold environment.

Several studies have suggested the contribution of gut microbiota to the host’s adaptation to cold environments [10, 14, 20]. For example, an increase in the relative abundance of the genus *Bacteroides* in the microbiota of Chinese alligators during hibernation contributes to the degradation of degrade host-derived mucin glycans [54]. However, the contribution of gut microbiota to cold adaptation may differ among host species or taxa [10]. *Phrynocephalus* lizards, 24 °C is a relatively low temperature at which they display poor physiological and behavioural performance [2]. In the 24 °C treatment, bacteria of the family Enterobacteriaceae were enriched in *P. erythrurus*, while Desulfovibrionaceae and *Clostridium sensu stricto 1* were enriched in *P. przewalskii* (Fig. 5). These bacteria had functional roles in fermentation and metabolism in both the species, probably contributing to their adaptation to cold environments. Generally, bacteria of the family Enterobacteriaceae have a role in glucose fermentation and reduce nitrates to nitrites [55], whereas Desulfovibrionaceae bacteria produce hydrogen sulfide through sulfate reduction [56], which increases the mucus layer permeability [57]. Bacteria of the genus *Clostridium sensu stricto 1* accelerate the use of cellulose in the host [58]. In conclusion, the gut bacteria that contribute to cold-climate tolerance differ significantly between the two *Phrynocephalus* lizards studied herein.

Similarly, the two species had different dominant bacteria at 30 °C (Figs. 2 and 5). Bacteria of the family Erysipelotrichaceae were more abundant in *P. erythrurus*, and those of the genera *Helicobacter* and the *Eubacterium coprostanoligenes group* were more abundant in *P. przewalskii* (Figs. 2 and 5). These bacteria play metabolism-related roles during digestion and absorption in both species, but their specific functions differed from 24 °C. For example, the family Erysipelotrichaceae is probably related to the host’s lipid metabolism and contributes to host nutrient absorption in *P. erythrurus* [59]. Meanwhile, the *Eubacterium coprostanoligenes group* family in *P. przewalskii* is correlated with the host lipid homeostasis [60]. In other known species, the gut microbiota in high-temperature environments is composed of mostly microbes harmful to their hosts [12, 24]. For example, the warm-adapted genus *Mycobacterium* exhibits higher abundances in *Rana pipiens* tadpoles reared at warm temperatures [42]. Meanwhile, the heat-induced changes in the gut microbiota in laying hens are associated with hepatic and intestinal dysfunction [12]. Compared with the findings reported for other animals, the two *Phrynocephalus* lizards we studied had gut microbiota better promoting thermal adaptation in cold environments.

Furthermore, the gene functions predicted based on the 16S rRNA sequences of gut microbiota in the two *Phrynocephalus* lizards were mainly metabolism-related, consistent with other known lizards [48, 61]. The gut microbes in *P. erythrurus* at 24 °C were primarily involved in environmental information processing and metabolism (including 12 KOs were mainly correlated with carbohydrate and salt metabolism) (Fig. 6). In contrast, the most significant gut microbial function in *P. przewalskii* at 24 °C, including two KOs, was related to only metabolism (Fig. 6). In addition, significant differences were detected between the two species in gut microbial function at 30 °C. The only KO related to metabolism was prominent at 30 °C in *P. erythrurus*, while the most significant function at 30 °C in *P. przewalskii* was related to amino acid metabolism (including three KOs) and cellular processes (including one KO) (Fig. 6). *Phrynocephalus erythrurus* at 24 °C had the highest relative abundance in the gut bacterial KOs than other species and temperature combinations, suggesting gut microbiota assists in long-term cold adaptation of *P. erythrurus* through high expression of metabolism-related genes. Above results indicate that gut microbiota from colder environments (*P. erythrurus*) had a higher proportion of metabolism-related functions than those from warmer environments (*P. przewalskii*), which might be more conducive to the host’s cold-climate adaptation. Thus, although the predicted gene functions are only the potential functions of gut bacteria, they reflect the response of gut microbiota to cold environments to some certain extent.

## Conclusion

In summary, this study shows that temperature affects the composition and relative abundance of the gut microbiota in the two *Phrynocephalus* lizards in a species-specific manner. Both species harbor specific gut microbiota with functional roles in cold-climate adaptation. Specifically, *P. erythrurus* have a higher Proteobacteria ratio and relative abundance of metabolism-related microbial genes in the gut than *P. przewalskii*. Given that *P. erythrurus* uses colder habitats than *P. przewalskii* all year round, these findings suggest that gut microbiota contributes to cold-climate adaptation in both species lizards but more evidently in *P. erythrurus*. This study provides evidence for the role of gut microbiota in cold adaptation. Future work focusing on the co-evolution mechanism between gut microbiota and their hosts in extreme environments will provide new insights into animal adaptation.

## Electronic supplementary material

Below is the link to the electronic supplementary material.

Supplementary Material 1

Supplementary Material 2

Supplementary Material 3

Supplementary Material 4

Supplementary Material 5

Supplementary Material 6

Supplementary Material 7

## Data Availability

The sequencing dataset for gut microbes has been submitted to the National Genomics Data Center (NGDC) GSA database (accession number CRA004548). The datasets supporting the conclusions of this article are also included within the article and its Additional files 1, 2, 3, and 4.

## Abbreviations

AAPS: Artificial atmospheric phenomena simulator.
ANOSIM: analysis of similarity.
ANOVA: Analysis of variance.
ASV: Amplicon sequence variant.
KEGG: Kyoto Encyclopedia of Genes and Genomes.
KO: KEGG Orthology.
LDA: Linear discriminatory analysis.
LEfSe: Linear discriminant analysis effect size.
NGDC: National Genomics Data Center.
PCoA: Principal coordinates analysis.
PCR: Polymerase chain reaction.
QIIME2: Quantitative Insights Into Microbial Ecology 2.
SE: Standard error.
