# Supplementary material for: Gut microbiota differs between two cold-climate lizards distributed in thermally different regions
Source: BMC Ecol Evol. 2022 Oct 21;22:120. doi: 10.1186/s12862-022-02077-8 (PMC9585762; doi:10.1186/s12862-022-02077-8)
Supplement: Supplementary file 1 — Supplementary Material 1 [file 12862_2022_2077_MOESM1_ESM.docx]

**Table S1** The number of valid reads of each fecal sample in two *Phrynocephalus* lizards treated with DADA2 and their number of biosamples in the National Genomics Data Center (NGDC) GSA database (accession number CRA004548)

| Sample ID | Species | Raw reads | Filtered | Denoised | Merged | Non-Chimeric  (Clean reads) | Biosample |
| --- | --- | --- | --- | --- | --- | --- | --- |
| A-1 | *P. przewalskii* | 94636 | 85217 | 84372 | 78465 | 67027 | SAMC432495 |
| A-3 | *P. przewalskii* | 88276 | 80178 | 78771 | 71339 | 56788 | SAMC432496 |
| A-4 | *P. przewalskii* | 85055 | 77164 | 76475 | 71937 | 64726 | SAMC432497 |
| A-6 | *P. przewalskii* | 78823 | 71472 | 70033 | 62850 | 53828 | SAMC432498 |
| A-7 | *P. przewalskii* | 96455 | 86961 | 85099 | 74568 | 59522 | SAMC432499 |
| A-10 | *P. przewalskii* | 97486 | 87805 | 86196 | 77735 | 66365 | SAMC432500 |
| B-1 | *P. przewalskii* | 105544 | 94919 | 93113 | 82650 | 71036 | SAMC432501 |
| B-3 | *P. przewalskii* | 110436 | 99306 | 96950 | 87170 | 69093 | SAMC432502 |
| B-4 | *P. przewalskii* | 107080 | 96387 | 94928 | 87327 | 79083 | SAMC432503 |
| B-6 | *P. przewalskii* | 94483 | 85350 | 84137 | 78957 | 69954 | SAMC432504 |
| B-7 | *P. przewalskii* | 106602 | 96362 | 94371 | 83589 | 72215 | SAMC432505 |
| B-10 | *P. przewalskii* | 90960 | 82679 | 82038 | 78562 | 74432 | SAMC432506 |
| A-60 | *P. przewalskii* | 107831 | 96420 | 94816 | 86266 | 77594 | SAMC432507 |
| A-63 | *P. przewalskii* | 104868 | 94881 | 93305 | 83276 | 68751 | SAMC432508 |
| A-64 | *P. przewalskii* | 87958 | 79262 | 77903 | 68070 | 58547 | SAMC432509 |
| A-65 | *P. przewalskii* | 107706 | 97009 | 96229 | 93653 | 89823 | SAMC432510 |
| A-66 | *P. przewalskii* | 99727 | 89396 | 87783 | 78380 | 68032 | SAMC432511 |
| A-67 | *P. przewalskii* | 100730 | 91053 | 89142 | 80157 | 66378 | SAMC432512 |
| A-68 | *P. przewalskii* | 99353 | 89100 | 88116 | 83854 | 76525 | SAMC432513 |
| A-71 | *P. przewalskii* | 98674 | 89076 | 88521 | 85351 | 81462 | SAMC432514 |
| B-60 | *P. przewalskii* | 74025 | 67294 | 66979 | 65547 | 64945 | SAMC432515 |
| B-63 | *P. przewalskii* | 110826 | 100086 | 98587 | 91047 | 82732 | SAMC432516 |
| B-64 | *P. przewalskii* | 107066 | 96372 | 94516 | 84431 | 70385 | SAMC432517 |
| B-65 | *P. przewalskii* | 97668 | 88274 | 87456 | 83416 | 80238 | SAMC432518 |
| B-66 | *P. przewalskii* | 97819 | 88168 | 87530 | 85825 | 78441 | SAMC432519 |
| B-67 | *P. przewalskii* | 111591 | 99772 | 99199 | 97340 | 93854 | SAMC432520 |
| B-68 | *P. przewalskii* | 95455 | 86147 | 84869 | 77897 | 63040 | SAMC432521 |
| B-71 | *P. przewalskii* | 98811 | 88688 | 87766 | 83507 | 74668 | SAMC432522 |
| A-17 | *P. erythrurus* | 71221 | 64769 | 63997 | 60093 | 56328 | SAMC432539 |
| A-20 | *P. erythrurus* | 84523 | 76312 | 75537 | 70434 | 64075 | SAMC432540 |
| A-21 | *P. erythrurus* | 96569 | 86953 | 86334 | 82639 | 74587 | SAMC432541 |
| A-22 | *P. erythrurus* | 87894 | 78600 | 78178 | 76303 | 74382 | SAMC432542 |
| A-23 | *P. erythrurus* | 99381 | 90232 | 89101 | 82382 | 60236 | SAMC432543 |
| A-26 | *P. erythrurus* | 42256 | 38480 | 38341 | 37937 | 35763 | SAMC432544 |
| B-17 | *P. erythrurus* | 132065 | 119467 | 118764 | 116013 | 113803 | SAMC432545 |
| B-18 | *P. erythrurus* | 125767 | 113229 | 112796 | 109904 | 103931 | SAMC432546 |
| B-20 | *P. erythrurus* | 92169 | 83482 | 83021 | 79372 | 63982 | SAMC432547 |
| B-22 | *P. erythrurus* | 91173 | 82751 | 82608 | 81747 | 74447 | SAMC432548 |
| B-23 | *P. erythrurus* | 104702 | 94259 | 94008 | 92585 | 81151 | SAMC432549 |
| B-26 | *P. erythrurus* | 111052 | 101040 | 100615 | 98503 | 93627 | SAMC432550 |
| A-47 | *P. erythrurus* | 90820 | 82951 | 81498 | 73498 | 59455 | SAMC432523 |
| A-48 | *P. erythrurus* | 95242 | 85755 | 84513 | 80820 | 73050 | SAMC432524 |
| A-49 | *P. erythrurus* | 103256 | 92950 | 91878 | 87018 | 77268 | SAMC432525 |
| A-51 | *P. erythrurus* | 87583 | 79454 | 78621 | 74974 | 69797 | SAMC432526 |
| A-53 | *P. erythrurus* | 95295 | 85937 | 85168 | 81316 | 74008 | SAMC432527 |
| A-54 | *P. erythrurus* | 99641 | 89162 | 88781 | 87209 | 81492 | SAMC432528 |
| A-56 | *P. erythrurus* | 73290 | 66501 | 64724 | 57150 | 44218 | SAMC432529 |
| A-58 | *P. erythrurus* | 94514 | 86121 | 85159 | 79567 | 73217 | SAMC432530 |
| B-47 | *P. erythrurus* | 97959 | 88372 | 86766 | 81055 | 72857 | SAMC432531 |
| B-48 | *P. erythrurus* | 92191 | 83001 | 81200 | 71753 | 63015 | SAMC432532 |
| B-49 | *P. erythrurus* | 145977 | 131849 | 129726 | 118204 | 100919 | SAMC432533 |
| B-52 | *P. erythrurus* | 95059 | 86105 | 85856 | 84604 | 78111 | SAMC432534 |
| B-53 | *P. erythrurus* | 90354 | 81779 | 81154 | 77857 | 66663 | SAMC432535 |
| B-54 | *P. erythrurus* | 105190 | 95533 | 95085 | 93027 | 90256 | SAMC432536 |
| B-55 | *P. erythrurus* | 93787 | 84650 | 84286 | 82837 | 76960 | SAMC432537 |
| B-56 | *P. erythrurus* | 94355 | 86068 | 85108 | 80186 | 75379 | SAMC432538 |
