# Supplementary material for: Gut microbiota differs between two cold-climate lizards distributed in thermally different regions
Source: BMC Ecol Evol. 2022 Oct 21;22:120. doi: 10.1186/s12862-022-02077-8 (PMC9585762; doi:10.1186/s12862-022-02077-8)
Supplement: Supplementary file 2 — Supplementary Material 2 [file 12862_2022_2077_MOESM2_ESM.docx]

**Table S2** The number of amplicon sequence variants (ASVs) of each fecal sample in two *Phrynocephalus* lizards. See Figure S1 for abbreviations for PP24, PP30, PE24, and PE30

| ***P. przewalskii*** | | |  | ***P. erythrurus*** | | |
| --- | --- | --- | --- | --- | --- | --- |
| Sample ID | Combination | ASVs |  | Sample ID | Combination | ASVs |
| A-1 | PP24 | 212 |  | A-17 | PE24 | 215 |
| A-3 | PP24 | 295 |  | A-20 | PE24 | 186 |
| A-4 | PP24 | 264 |  | A-21 | PE24 | 179 |
| A-6 | PP24 | 300 |  | A-22 | PE24 | 147 |
| A-7 | PP24 | 315 |  | A-23 | PE24 | 177 |
| A-10 | PP24 | 338 |  | A-26 | PE24 | 58 |
| B-1 | PP24 | 371 |  | B-17 | PE24 | 251 |
| B-3 | PP24 | 338 |  | B-18 | PE24 | 218 |
| B-4 | PP24 | 308 |  | B-20 | PE24 | 111 |
| B-6 | PP24 | 309 |  | B-22 | PE24 | 56 |
| B-7 | PP24 | 363 |  | B-23 | PE24 | 54 |
| B-10 | PP24 | 363 |  | B-26 | PE24 | 123 |
| A-60 | PP30 | 371 |  | A-47 | PE30 | 171 |
| A-63 | PP30 | 355 |  | A-48 | PE30 | 222 |
| A-64 | PP30 | 337 |  | A-49 | PE30 | 237 |
| A-65 | PP30 | 255 |  | A-51 | PE30 | 194 |
| A-66 | PP30 | 338 |  | A-53 | PE30 | 155 |
| A-67 | PP30 | 316 |  | A-54 | PE30 | 176 |
| A-68 | PP30 | 226 |  | A-56 | PE30 | 198 |
| A-71 | PP30 | 266 |  | A-58 | PE30 | 224 |
| B-60 | PP30 | 287 |  | B-47 | PE30 | 220 |
| B-63 | PP30 | 366 |  | B-48 | PE30 | 234 |
| B-64 | PP30 | 351 |  | B-49 | PE30 | 265 |
| B-65 | PP30 | 317 |  | B-52 | PE30 | 44 |
| B-66 | PP30 | 272 |  | B-53 | PE30 | 138 |
| B-67 | PP30 | 147 |  | B-54 | PE30 | 241 |
| B-68 | PP30 | 259 |  | B-55 | PE30 | 229 |
| B-71 | PP30 | 274 |  | B-56 | PE30 | 234 |
