# Supplementary material for: Gut microbiota differs between two cold-climate lizards distributed in thermally different regions
Source: BMC Ecol Evol. 2022 Oct 21;22:120. doi: 10.1186/s12862-022-02077-8 (PMC9585762; doi:10.1186/s12862-022-02077-8)
Supplement: Supplementary file 3 — Supplementary Material 3 [file 12862_2022_2077_MOESM3_ESM.docx]

**Table S3** The relative abundance of unique bacterial taxon among different groups based on Kruskal-Wallis test. The letters “p”, “c”, “o”, “f” and “g” indicate phylum, class, order, family and genus, respectively

| Taxonomy | *df* | *H* | *p* |
| --- | --- | --- | --- |
| p__Desulfobacterota | 3 | 18.915 | 0.000 |
| p__Firmicutes | 3 | 10.878 | 0.012 |
| p__Campilobacterota | 3 | 13.516 | 0.004 |
| c__Campylobacteria | 3 | 4.545 | 0.208 |
| c__Desulfovibrionia | 3 | 10.119 | 0.018 |
| c__Clostridia | 3 | 16.165 | 0.001 |
| c__Bacilli | 3 | 26.695 | 0.000 |
| o__Clostridiales | 3 | 14.794 | 0.002 |
| o__Campylobacterales | 3 | 7.273 | 0.064 |
| o__Erysipelotrichaleles | 3 | 16.427 | 0.001 |
| o__Desulfovibrionaleles | 3 | 16.130 | 0.001 |
| o__Enterobacterales | 3 | 12.469 | 0.006 |
| o__Bacillales | 3 | 16.451 | 0.001 |
| o__Oscillospirales | 3 | 18.968 | 0.000 |
| o__Lactobacillales | 3 | 21.777 | 0.000 |
| f__Oscillospiraceae | 3 | 20.320 | 0.000 |
| f__Rikenellaceae | 3 | 12.202 | 0.007 |
| f__Desulfovibrionaceae | 3 | 18.915 | 0.000 |
| f__Helicobacteraceae | 3 | 13.516 | 0.004 |
| f__Erysipelotrichaceae | 3 | 15.173 | 0.002 |
| f__Enterobacteriaceae | 3 | 15.905 | 0.001 |
| f__Ruminococcaceae | 3 | 12.850 | 0.005 |
| f__[Eubacterium]_coprostanoligenes_group | 3 | 24.280 | 0.000 |
| f__Enterococcaceae | 3 | 19.352 | 0.000 |
| f__Clostridiaceae | 3 | 16.885 | 0.001 |
| f__Bacillaceae | 3 | 18.867 | 0.000 |
| g__*Alistipes* | 3 | 1.676 | 0.642 |
| g__*Tannerellaceae* | 3 | 2.553 | 0.466 |
| g__*Helicobacter* | 3 | 10.534 | 0.015 |
| g__*Eubacterium_coprostanoligenes_group* | 3 | 7.923 | 0.0478 |
| g__*Enterococcus* | 3 | 5.4002 | 0.145 |
| g__*NK4A214_group* | 3 | 2.467 | 0.481 |
| g__*Rikenella* | 3 | 0.561 | 0.905 |
| g__*Caproiciproducens* | 3 | 1.951 | 0.583 |
| g__*Clostridium_sensu_stricto_1* | 3 | 11.096 | 0.011 |
| g__*Tyzzerella* | 3 | 2.308 | 0.511 |
| g__*Bacillus* | 3 | 1.675 | 0.642 |
