# Supplementary material for: Gut microbiota differs between two cold-climate lizards distributed in thermally different regions
Source: BMC Ecol Evol. 2022 Oct 21;22:120. doi: 10.1186/s12862-022-02077-8 (PMC9585762; doi:10.1186/s12862-022-02077-8)
Supplement: Supplementary file 4 — Supplementary Material 4 [file 12862_2022_2077_MOESM4_ESM.docx]

Table S4 The relative abundance of unique predicated functions among different groups based on Kruskal-Wallis test

| Level name | *df* | *H* | *p* |
| --- | --- | --- | --- |
| cell process | 3 | 12.772 | 0.0048 |
| Cell_motility | 3 | 10.576 | 0.014 |
| Environmental Information Processing | 3 | 9.061 | 0.028 |
| Membrane transport | 3 | 11.601 | 0.009 |
| Amino acid metabolism | 3 | 18.319 | 0.000 |
| ko00450 | 3 | 17.428 | 0.001 |
| ko00540 | 3 | 11.762 | 0.008 |
| ko00440 | 3 | 9.739 | 0.021 |
| ko00051 | 3 | 15.445 | 0.001 |
| ko00473 | 3 | 16.088 | 0.001 |
| ko01055 | 3 | 9.614 | 0.022 |
| ko00040 | 3 | 5.375 | 0.146 |
| ko00020 | 3 | 10.927 | 0.012 |
| ko00633 | 3 | 15.636 | 0.001 |
| ko02030 | 3 | 12.610 | 0.006 |
| ko00500 | 3 | 9.454 | 0.024 |
| ko00052 | 3 | 9.477 | 0.024 |
| ko00920 | 3 | 16.745 | 0.001 |
| ko00561 | 3 | 15.534 | 0.001 |
| ko00591 | 3 | 13.970 | 0.003 |
| ko01053 | 3 | 19.936 | 0.000 |
| ko00430 | 3 | 15.156 | 0.002 |
| ko00130 | 3 | 16.135 | 0.001 |
