# Supplementary figures and images for: Gut microbiota differs between two cold-climate lizards distributed in thermally different regions
Source: BMC Ecol Evol. 2022 Oct 21;22:120. doi: 10.1186/s12862-022-02077-8 (PMC9585762; doi:10.1186/s12862-022-02077-8)

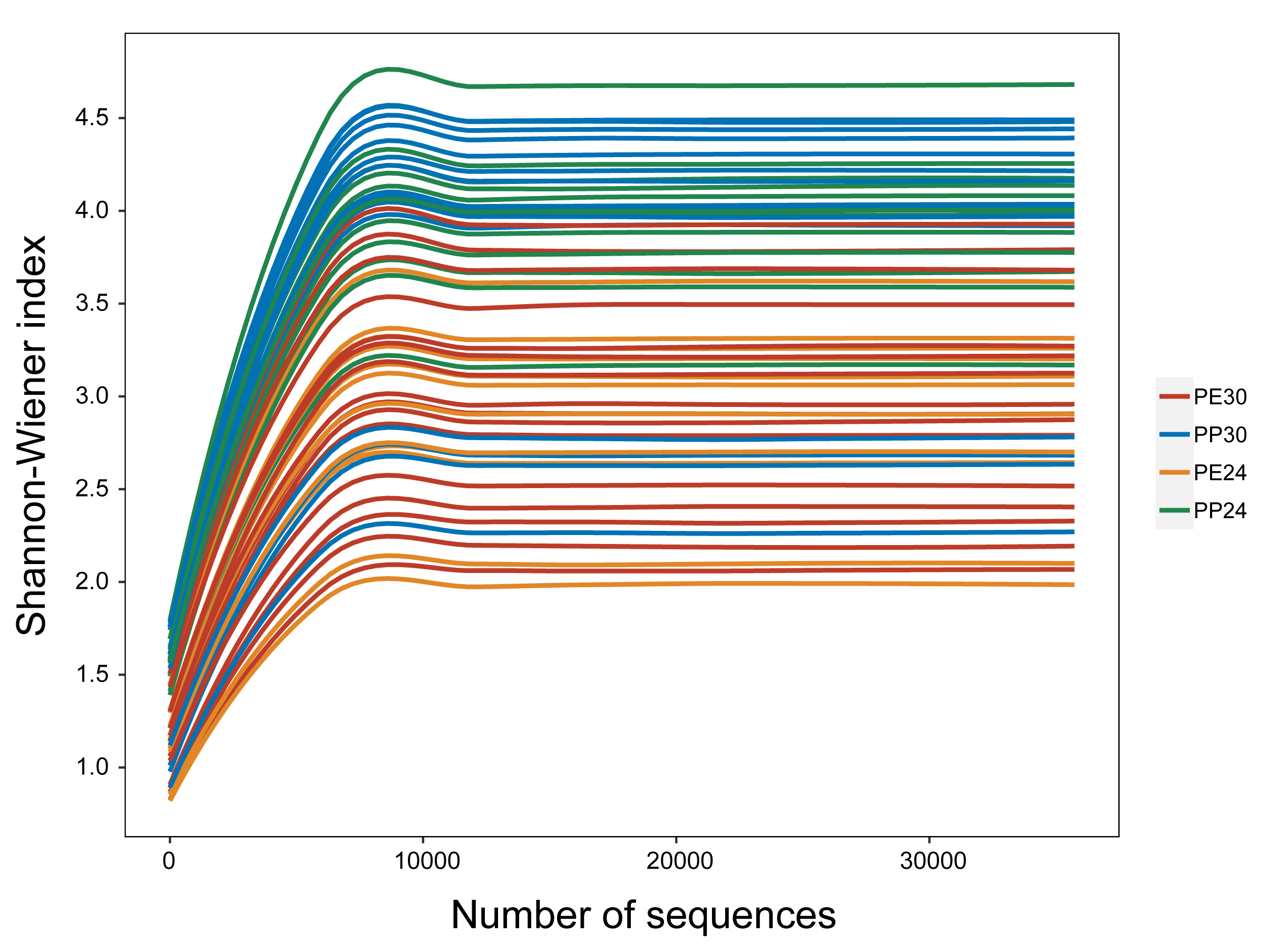

Supplement: Supplementary file 5 — Supplementary Material 5 [file 12862_2022_2077_MOESM5_ESM.png]

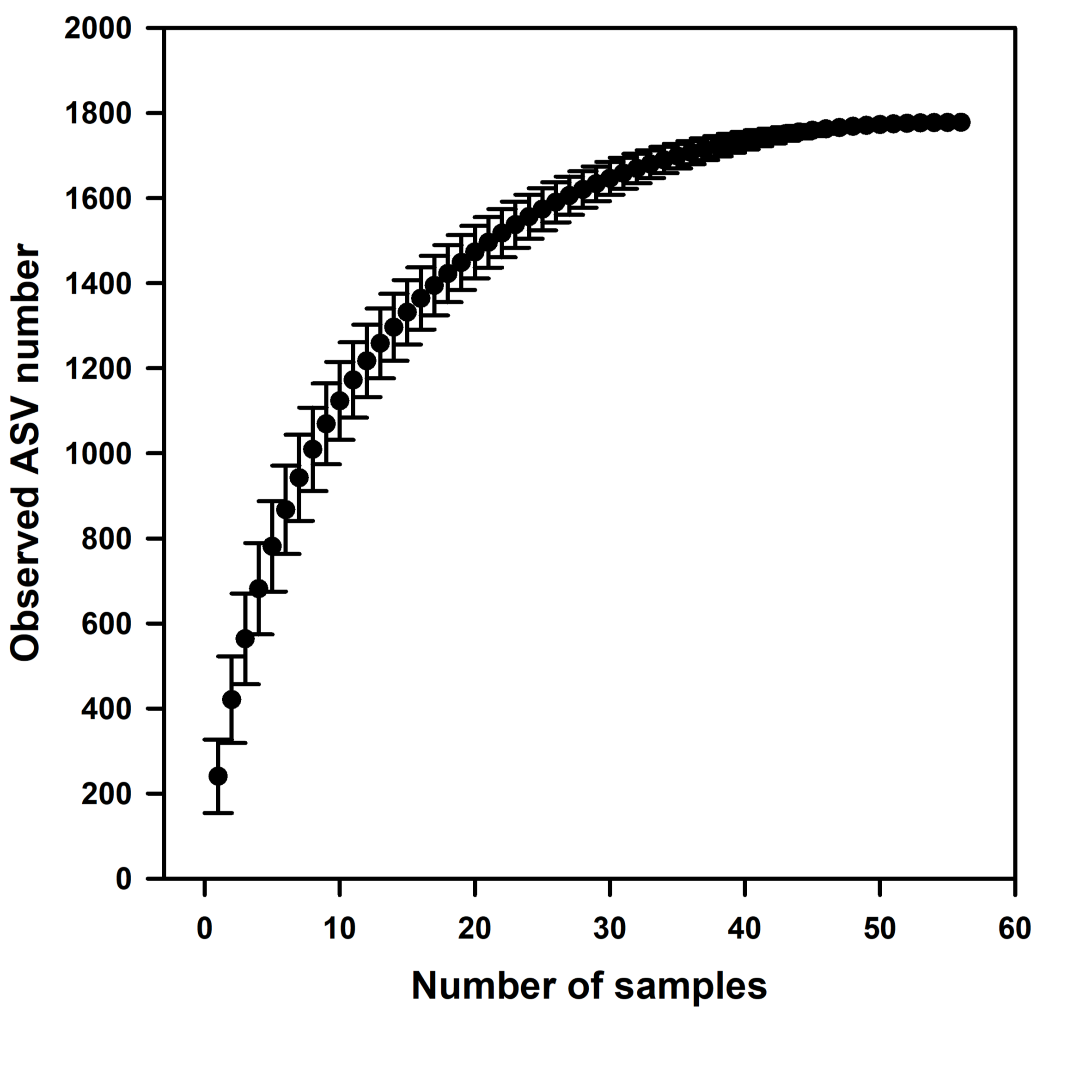

Supplement: Supplementary file 6 — Supplementary Material 6 [file 12862_2022_2077_MOESM6_ESM.png]

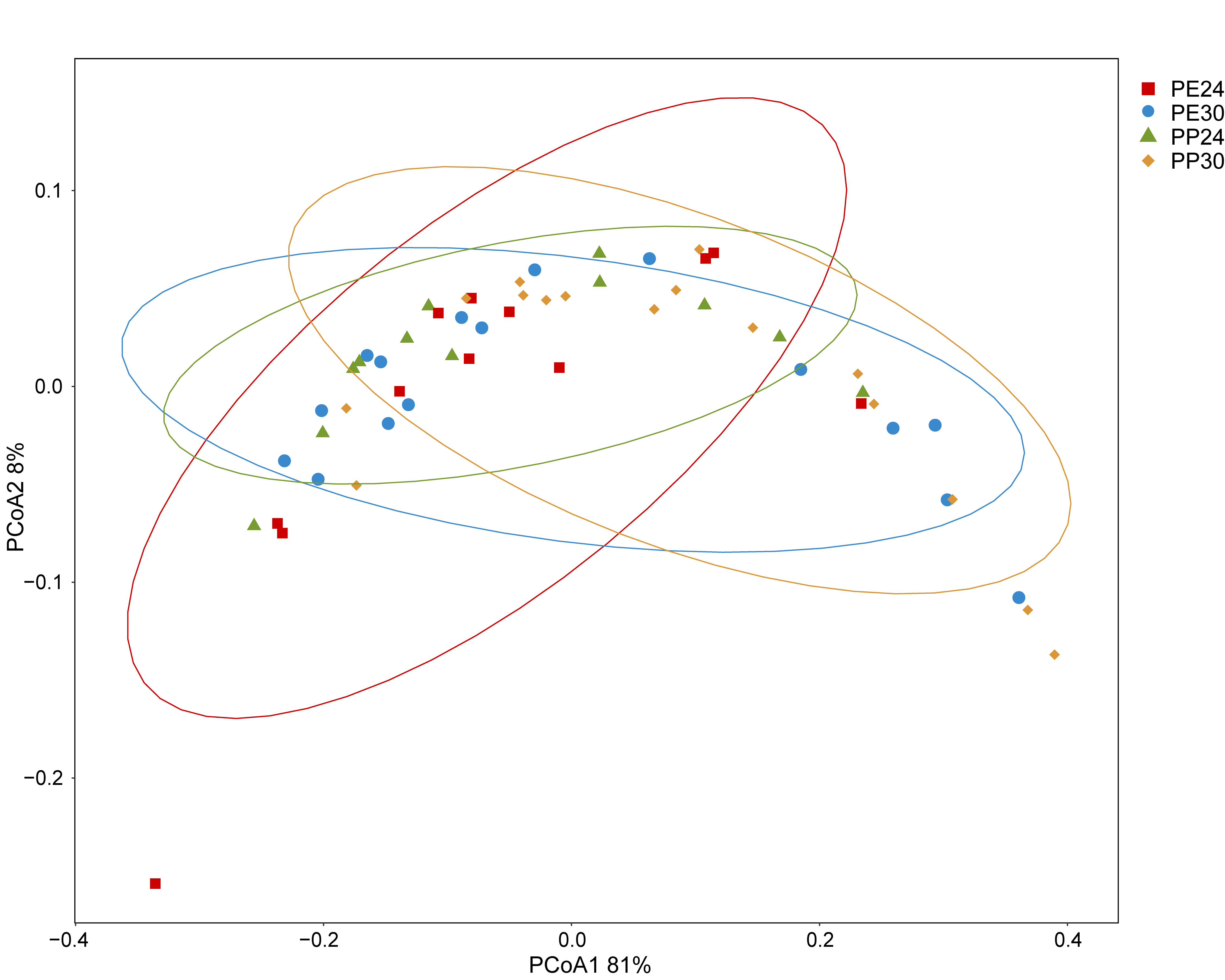

Supplement: Supplementary file 7 — Supplementary Material 7 [file 12862_2022_2077_MOESM7_ESM.png]
